# Characteristics and immune checkpoint inhibitor effects on non-smoking non-small cell lung cancer with *KRAS* mutation: A single center cohort (STROBE-compliant)

**DOI:** 10.1097/MD.0000000000029381

**Published:** 2022-06-17

**Authors:** Jia-Jun Wu, Po-Hsin Lee, Zhe-Rong Zheng, Yen-Hsiang Huang, Jeng-Sen Tseng, Kuo-Hsuan Hsu, Tsung-Ying Yang, Sung-Liang Yu, Kun-Chieh Chen, Gee-Chen Chang

**Affiliations:** a Division of Chest Medicine, Department of Internal Medicine, Taichung Veterans General Hospital, Taichung, Taiwan; b Taipei Veterans General Hospital, Taoyuan Branch, Taoyuan, Taiwan; c Division of Pulmonary Medicine, Department of Internal Medicine, Chung Shan Medical University Hospital, Taichung, Taiwan; d School of Medicine, Chung Shan Medical University, Taichung, Taiwan; e Institute of Medicine, Chung Shan Medical University, Taichung, Taiwan; f College of Medicine, National Yang Ming Chiao Tung University, Taipei, Taiwan; g Ph.D. Program in Translational Medicine, National Chung Hsing University; h Rong Hsing Research Center For Translational Medicine, National Chung Hsing University; i Institute of Biomedical Sciences, National Chung Hsing University, Taichung, Taiwan; j Faculty of Medicine, School of Medicine, National Yang Ming Chiao Tung University, Taipei, Taiwan; k Department of Post-Baccalaureate Medicine, College of Medicine, National Chung Hsing University, Taichung, Taiwan; l Division of Critical Care and Respiratory Therapy, Department of Internal Medicine, Taichung Veterans General Hospital, Taichung, Taiwan; m Department of Life Sciences, National Chung Hsing University, Taichung, Taiwan; n Department of Clinical and Laboratory Sciences and Medical Biotechnology, National Taiwan University College of Medicine, Taipei, Taiwan; o Department of Applied Chemistry, National Chi Nan University, Nantou, Taiwan.

**Keywords:** immune checkpoint inhibitor, Kirsten rat sarcoma, lung cancer, never-smoker, programmed death-ligand 1

## Abstract

Kirsten rat sarcoma (*KRAS*) mutation (*KRASm*) is associated with poor prognosis in non-small cell lung cancer (NSCLC) patients. We have aimed to survey NSCLC patients harboring *KRASm* in Taiwan, where never-smoking lung adenocarcinoma predominates, and analyze the immune checkpoint inhibitor effect on NSCLC harboring *KRASm*.

NSCLC patients with *KRASm* were enrolled and tested on programmed death-ligand 1 (PD-L1) expression using available tissue. We analyzed their clinical features, PD-L1 status, responses to ICIs, and overall survival (OS).

We studied 93 patients with a median age 66.0 years, 23.7% of whom were women, and 22.6% were never-smokers. The results showed that G12C (36.6%) was the most common *KRASm*. In 47 patients with available tissue for PD-L1 testing, PD-L1 expression was positive in 66.0% of patients, while PD-L1 ≥50% was higher in ever-smokers (*P* = .038). Among 23 patients receiving ICI treatment, those with PD-L1 ≥50% experience a 45.5% response rate to ICI. There were benefits from ICI treatment on OS compared with no ICI treatment (median OS 35.6 vs 9.8 months, *P* = .002) for all of our patients, and for patients with PD-L1 ≥50% (median OS not-reached vs 8.4 months, *P* = .008). There were no differences in survival across different KRAS subtypes (*P* = .666).

Never-smokers composed more than one-fifth of *KRASm* in NSCLC in Taiwan. A high PD-L1 expression was related to smoking history and responded well to ICI. ICI treatment improved the OS in NSCLC patients with KRASm, particularly those with PD-L1 ≥50%.

## 1 Introduction

Regardless of gender, lung cancer is the leading cause of cancer-related deaths worldwide.^[1]^ Kirsten rat sarcoma (*KRAS*) mutation have been the most common driver gene mutation in non-small cell lung cancers (NSCLC) worldwide, accounted for 20% to 25% in lung adenocarcinoma.^[2–4]^ In Taiwan, however, *KRAS* mutation was found in only 3.3% to 5.0% of patients, while the epidermal growth factor receptor (*EGFR*) and as well as the rearrangement of the anaplastic lymphoma kinase (*ALK*) gene were more common.^[5,6]^

NSCLC patients with *KRAS* mutation have a notoriously poor prognosis.^[6–8]^ Although target agents against *KRAS* G12C mutation, such as sotorasib, have been revealed promising efficacy, the unmet need of the treatment for other *KRAS* subtypes remained unresolved.^[9]^ On the other hand, immune checkpoint inhibitor (ICI) may provide survival benefits for NSCLC patients harboring the *KRAS* mutation.^[10,11]^ Factors such as the expression level of programmed death-ligand 1 (PD-L1) or different *KRAS* subtypes may predict the ICI treatment outcomes, but it remains unclear.^[12]^

*KRAS* mutations are strongly associated with smoking, and with heterogeneous oncogenic substitutions. G12C were the most common subtype in lung adenocarcinoma.^[2]^ Smoking habits are known to affect incidences of the different subtypes, and may contribute to different clinical outcomes.^[13,14]^ In Taiwan, more than half (53%) of lung cancer patients were never smokers, with lung adenocarcinoma being the major histological type.^[15]^ In the present study, we aimed to characterize the clinical and pathological features of patients with *KRAS* mutation in this non-smoker predominant area. In addition, we compared the differences between never smokers and ever/current smokers, including *KRAS* subtypes, PD-L1 expression, ICI responsiveness, and survivals.

## 2 Material and methods

### 2.1 Study design

Patients were selected retrospectively at Taichung Veterans General Hospital from April 2011 to March 2020. Treatment-naïve non-small cell lung cancer patients with tumor specimens were eligible for initial screening. Patients with lung adenocarcinoma, or TTF-1 positive NSCLC were eligible for the genetic study. For non-adenocarcinoma, or TTF-1 negative NSCLC patients, only never smokers qualified. Regarding *KRAS* mutation study, which intended to evaluate the effects of ICI therapy, we excluded patients who were not confirmed to be primary lung cancer histologically. Additionally, it excluded those who had active cancer in other sites simultaneously; being either stage I–IIIA or incomplete staging; poor performance status (Eastern Cooperative Oncology Group Performance Status 3–4); receiving either hospice care or no treatment after diagnosis; as well as those who had received *KRAS*-targeted therapy. Our study was approved by the Institutional Review Board of Taichung Veterans General Hospital (IRB No. C08197, CF12019, and CF15271).

### 2.2 Identification of driver mutations and PD-L1 assay

Tumor specimens were procured for analyses of mutations of genes, including *EGFR*, *KRAS*, human epidermal growth factor 2 (*HER2*), v-raf murine sarcoma viral oncogene homolog B (*BRAF*), and *ALK*, and programmed death-ligand 1 (PD-L1) with IHC assays as previously described.^[5,16]^ DNA was extracted from tumors using a QIAmp DNA Mini kit (Qiagen, Valencia, CA) following the manufacturer's instructions. *EGFR*, *KRAS*, *HER2*, and *BRAF* mutations were assessed through matrix-assisted laser desorption ionization-time of flight mass spectrometry (MALDI-TOF MS).^[5]^ MassARRAY analyses were performed following the manufacturer's instructions.

Lung adenocarcinoma with *KRAS* mutations consists of single amino acid substitutions in hotspots located mostly in codon 12 and less frequently in codons 13 and 61.^[17,18]^ In this study, we only detected mutations in codons 12 and 13 as previously described.^[5]^*ALK* translocation was detected using the Ventana method. All tests were performed by the ISO15189-certified TR6 Pharmacogenomics Lab (PGL), the National Research Program for Biopharmaceuticals (NRPB), and the National Center of Excellence for Clinical Trial and Research of NTUH.

One of the commercial PD-L1 IHC assays involving either 22C3, or SP263, was performed for patients who provided adequate specimens. Among them, the PD-L1 IHC 22C3 pharmDx were conducted on the DAKO Autostainer Link 48, while the Ventana PD-L1 SP263 assay was conducted on the Ventana BanchMark platform. All histological slides were peer reviewed by 2 pathologists who had attended international training workshops conducted by Agilent Technologies Inc./DAKO Corp and Roche/Ventana Medical Systems Inc., regarding the detection of PD-L1 immunoreaction. The PD-L1 expression were defined as tumor proportion score (TPS), which was the ratio between stained tumor cell and viable tumor cells.^[19]^ The results were concurred in the intradepartmental consensus meeting.

### 2.3 Data records and response evaluation

Clinical data of patients included age, gender, Eastern Cooperative Oncology Group performance status, tumor stage, and smoking status (never-smokers were defined as those who had never smoked a single cigarette, whereas ever-smokers were defined as those currently smoking or had formerly smoked). TNM (tumor, node, and metastases) staging followed the 8th edition of the American Joint Committee for Cancer (AJCC) staging system.^[20]^ Response assessment followed the Response Evaluation Criteria in Solid Tumors (RECIST) version 1.1.

### 2.4 Endpoints

First, we compared the clinicopathological characteristics of non-smoked and ever-smoked patients with *KRAS* mutations NSCLC. We also studied the distribution of different *KRAS* subtypes and their influences on disease outcomes, including the response rate to ICIs and survival outcomes.

Second, we aimed to analyze the PD-L1 expression and the effect of ICI treatment in *KRAS* patients. Patients who received 1 cycle or more of ICIs during the follow-up period were classified as the ICI treatment group. Patients who did not undergo any ICI treatment during follow-up were regarded as the non-ICI treatment group.

### 2.5 Statistical methods

The Chi-square test, Mantel–Haenszel test, paired independent sample *t* test, Mann–Whitney *U* test, one-way analysis of variance, and logistic regression were all used to compare inter-group differences with respect to the categorical and continuous variables wherever appropriate; a *P* value <.05 was with significant difference. Overall survival (OS) was measured as being the time from disease diagnosis to death due to any reason. Patients were censored if alive at the time of analysis during the last follow-up. OS was estimated using the Kaplan–Meier method, whereas the inter-group difference in OS was assessed using the stratified log-rank test. The Cox proportional hazard model for multivariate analyses was used to evaluate OS. Two-tailed tests with *P* values of <.05 were considered statistically significant.

All analyses were performed using the IBM SPSS Statistics package, version 23 (IBM Corporation, Armonk, NY).

## 3 Results

### 3.1 Patient characteristics and clinicopathological presentations

A total of 2932 patients from a single medical center were tested for the 5 driver genes described above (see Table S1, Supplemental Digital Content 1, which revealed patients tested for 5 driver genes), with 151 (5.2%) patients having *KRAS* mutation. After excluding 58 of those patients who were not deemed fit for evaluating the effects of ICI therapy, a total of 93 patients were enrolled for analysis (Figure S1, see Supplemental Digital Content 2, which demonstrated the flow chart of patient enrollment). The descriptive characteristics of the patients are summarized in Table 1, with their median age being 66.0 years. Amongst them, 22 (23.7%) were women and 21 (22.6%) were never-smokers. Adenocarcinoma was diagnosed in 87 patients, squamous cell carcinoma in 2, and other cell types in 3. Among the *KRAS* mutation patients, we found only a few other co-driver mutations, that is, 1 *EGFR* co-mutation (exon 19 deletion), 1 *ALK* translocation, and 1 *BRAF V600E* co-mutation. We found no co-mutations with *HER2* exon 20 insertion (Table 1). Compared with ever or current smoker, those who never smoked were older in age (71.0 vs 63.0, *P* = .020), and with more female cases (66.7% vs 11.1%, *P* < .001).

**Table 1:** Demographic data.

	All (n = 93)	Never smoker (n = 21)	Ever smoker (n = 72)	*P* value^∗^
Age, medium (IQR)	66.0 (56.0–72.0)	71.0 (62.5–77.0)	63.0 (56.0–71.0)	.020
Gender, number (%)				
Male	71 (76.3)	7 (33.3)	64 (88.9)	<.001
Female	22 (23.7)	14 (66.7)	8 (11.1)	
Stage^a^, number (%)				
IIIB–IIIC	7 (7.6)	0 (0)	7 (9.7)	.344
IVA–IVB	86 (92.4)	21 (100.0)	65 (90.3)	
ECOG PS				
0–1	80 (86.0)	16 (76.2)	64 (88.9)	.160
2	13 (14.0)	5 (23.8)	8 (11.1)	
Pathology, number (%)				
Adenocarcinoma	87 (93.5)	19 (90.5)	68 (94.4)	.711
Invasive mucinous adenocarcinoma	1 (1.1)	0 (0)	1 (1.4)	
Squamous cell carcinoma	2 (2.2)	1 (4.8)	1 (1.4)	
Others^b^	3 (3.2)	1 (4.8)	2 (2.8)	
*KRAS* mutation subtype				
G12C	34 (36.6)	6 (28.6)	28 (38.9)	.449
Non-G12C	59 (63.4)	15 (71.4)	44 (61.1)	
Driver gene mutation other than *KRAS*				
No co-mutation	90 (96.8)	20 (95.2)	70 (97.2)	.259
*EGFR*, number (%)^c^	1 (1.1)	0 (0)	1 (1.4)	
*ALK*, number (%)	1 (1.1)	1 (4.8)	0 (0)	
*HER2*, number (%)	0 (0)	0 (0)	0 (0)	
*BRAF*, number (%)	1 (1.1)	0 (0)	1 (1.4)	

*ALK* = anaplastic lymphoma kinase, *BRAF* = v-raf murine sarcoma viral oncogene homolog B, ECOG PS = Eastern Cooperative Oncology Group performance status, *EGFR* = Epidermal growth factor receptor, *HER2* = human epidermal growth factor 2, *KRAS* = Kirsten rat sarcoma.

a AJCC 8th edition.

b Two adenosquamous carcinoma, 1 pleomorphic carcinoma.

c *Del*19.

∗ Probability value compared by Mann-Whitney U test or Chi-square test.

**Figure 1:**
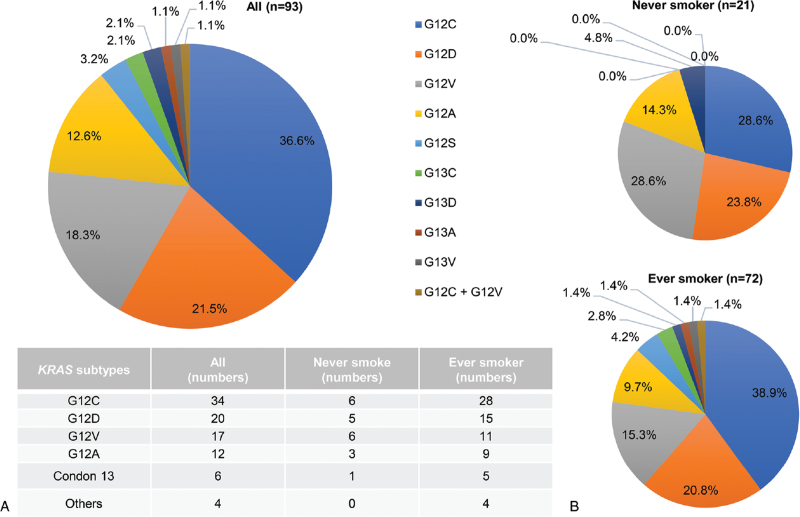
The distribution of *KRAS* mutation subtypes of all patients (A), never or ever smoker (B). G12C remained the most common *KRAS* mutation subtype in both the ever and never smokers. *KRAS* = Kirsten rat sarcoma.

We further analyzed the distribution of different *KRAS* subtypes (Fig. 1). In particular, we detected those mutations in both codon 12 and 13. The mutation rate in codon 12 was much higher than in codon 13, that is, 87 patients (93.5%) versus 6 patients (6.4%), respectively. G12C was the most common subtype of *KRAS* mutation (36.6%). Ever smokers tended to be with more G12C than never smokers, although the difference was not significant (*P* = .449) (Table 1; see Table S2, Supplemental Digital Content 3, which revealed smoking behavior across different genders and *KRAS* subtypes).

### 3.2 PD-L1 expression and the response to immune checkpoint inhibitor (ICI)

PD-L1 expression analyses were performed on 47 patients, where positive PD-L1 results were found in 31 (66.0%) patients, with 18 (38.3%) of them showing high expression levels (TPS ≥50%) (Table 2; see Figure S2A, Supplemental Digital Content 4, which demonstrated the distribution of PD-L1 expression). Ever-smokers were more likely to have TPS ≥50% than those who never smoked (*P* = .038). Factors including age, gender, and *KRAS* subtypes had no impacts on PD-L1 expression.

**Table 2:** Characteristics for patients who had PD-L1 results (n = 47).

	All	TPS <1%	TPS 1–49%	TPS ≥50%	*P* value^∗^
N	47	16 (34.0)	13 (27.7)	18 (38.3)	
Age, medium (IQR)	67.0 (56.0–72.0)	61.5 (56.6–72.0)	64.0 (53.0–73.5)	67.5 (55.8–71.0)	.937
Gender, number (%)					
Male	31	11 (35.5)	7 (22.6)	13 (41.9)	.543
Female	16	5 (31.3)	6 (37.5)	5 (31.1)	
Smoking, number (%)					
Never smoke	14	7 (50.0)	5 (35.7)	2 (14.3)	.038
Ever smoke	33	9 (27.3)	8 (24.2)	16 (48.5)	
*KRAS* subtype, number (%)					
G12C	15	5 (33.3)	5 (33.3)	5 (33.3)	.818
Non-G12C	32	11 (34.4)	8 (25.0)	13 (40.6)	

ICI = immune check point inhibitor, *KRAS* = Kirsten rat sarcoma, N = number of patients, PD-L1 = programmed death-ligand 1, TPS = tumor proportion score.

∗ Probability value compared by Mann-Whitney U test or Chi-square test.

ICI treatments were administered to 23 patients (Table 3; see Table S3, Supplemental Digital Content 5, which revealed patients who received immunotherapy; see Figure S2B, Supplemental Digital Content 4, which demonstrated the responses to ICI treatment). Partial response was found in 5 (21.7%) of the patients, stable disease in 4 (17.4%), and disease progression in 14 (60.9%). Never smokers showed a similar response to ICI as those who ever smoked. Patients with TPS ≥50% were more responsive to ICI, with a response rate of 45.5% (*P* = .035).

**Table 3:** Characteristics for patients who had ICI treatments (n = 23).

	All	PR	SD	PD	*P* value^∗^
N	23	5 (21.7)	4 (17.4)	14 (60.9)	
Age, medium (IQR)	57.0 (51.0–68.0)	68.0 (53.0–72.5)	55.5 (50.0–67.8)	56.5 (50.8–61.8)	.510
Gender, number (%)					
Male	16	2 (12.5)	4 (25.0)	10 (62.5)	.147
Female	7	3 (42.9)	0 (0)	4 (57.1)	
Smoking, number (%)					
Never smoker	3	0 (0)	0 (0)	3 (100)	.330
Ever smoke	20	5 (25.0)	4 (20.0)	11 (55.0)	
TPS, number (%)					
≥50%	11	5 (45.5)	1 (9.1)	5 (45.5)	.035
<1%, or 1–49%	11	0 (0)	3 (27.3)	8 (72.7)	
*KRAS* subtype, number (%)					
G12C	7	2 (28.6)	2 (28.6)	3 (42.9)	.478
Non-G12C	16	3 (18.8)	2 (12.5)	11 (68.8)	

ICI = immune check point inhibitor, *KRAS* = Kirsten rat sarcoma, N = number of patients, PD = disease progression, PR = partial response, SD = stable disease, TPS = tumor proportion score.

∗ Probability value compared by Mann-Whitney U test or Chi-square test.

### 3.3 Patient survivals

**Figure 2:**
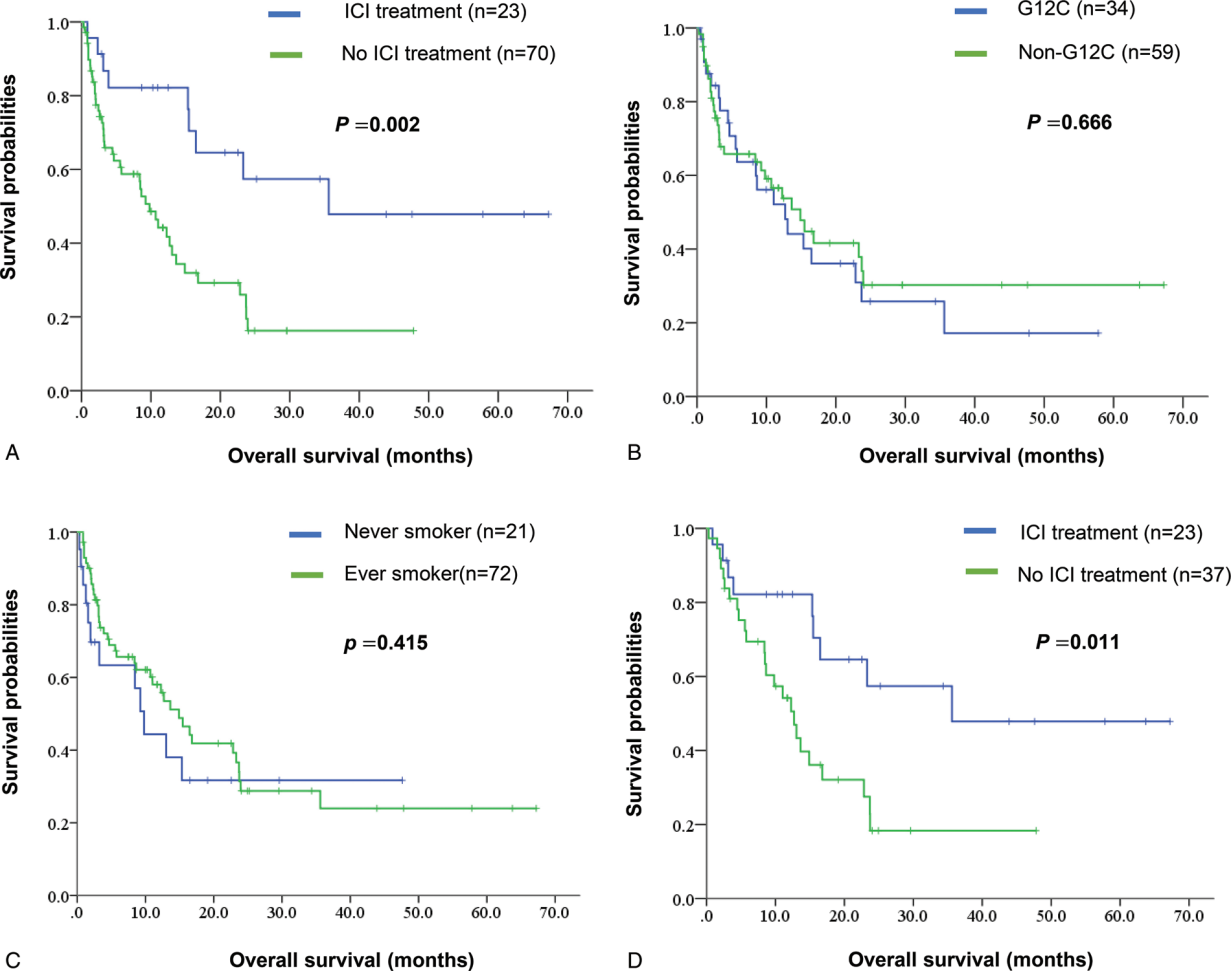
Overall survival (OS) by the Kaplan–Meier methods comparing the use of ICI treatment or not in all patients (A), comparing OS across different *KRAS* subtypes (B), comparing OS between never smokers and ever smokers (C), and comparing the use of ICI treatment or not after excluding those who did not receive second-line therapy (D). ICI treatment shows longer OS for all (median OS 35.6 vs 9.8 months) or after adjustment (median OS 35.6 vs 12.7 months). There was no difference in OS across the different *KRAS* subtypes or smoking behaviors. ICI = immune check point inhibitor, *KRAS* = Kirsten rat sarcoma.

Among our patients, the median OS was 13.0 months overall. Patients who underwent ICI treatments displayed a significantly better OS than who did not, with median OS being 35.6 (15.5–NR) versus 9.8 (7.1–12.5) months, respectively (*P* = .002) (Fig. 2A). Neither the smoking history, nor the *KRAS* mutation subtypes showed a significant difference on patients’ survival (Fig. 2B and C). For those who did not receive immunotherapy, there were 33 patients did not have second line treatment and chose hospice care. After adjusted for those who did not complete second line treatment, ICI therapy still showed significant benefit on the survival, with median OS 35.6 (15.5–NR) versus 12.7 (8.7–16.7) months (*P* = .011) (Fig. 2D).

**Figure 3:**
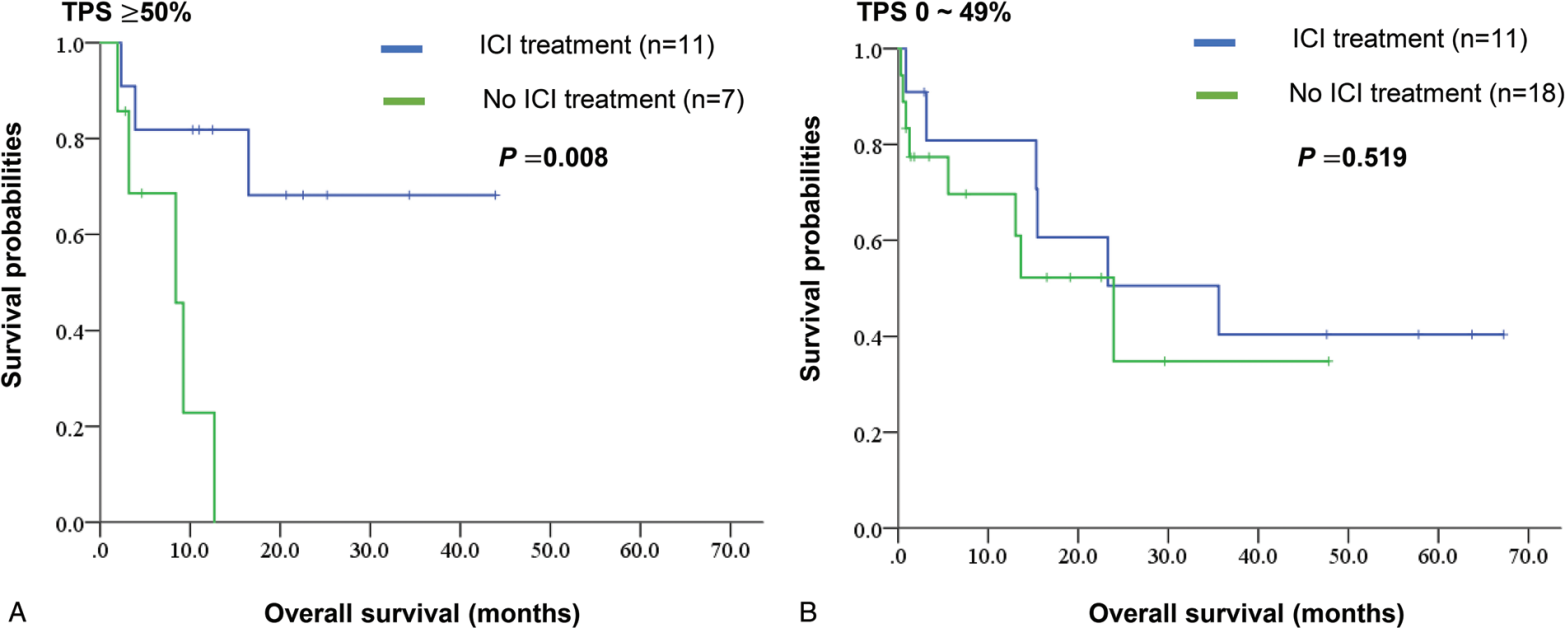
Overall survival (OS) by the Kaplan–Meier methods comparing the effect of ICI treatment on patients with high PD-L1 (TPS ≥50%) (A), and patients with low or negative PD-L1 (TPS 1–49% or <1%) (B). ICI treatment shows longer OS for patients with TPS ≥50% (median OS not reach vs 8.4 months), but offered no obvious effect on survival for patients with TPS 0–49% (median OS 35.6 vs 23.9 months). ICI = immune check point inhibitor, *KRAS* = Kirsten rat sarcoma, PD-L1 = programmed death-ligand 1, TPS = tumor proportion score.

In addition, we did an analysis for survival of patients with PD-L1 results. For patients with a TPS ≥50%, the ICI treatment group experienced better survival, with median OS not-reached versus 8.4 (2.6–14.2) months (*P* = .008) (Fig. 3A). ICI treatment offered no survival benefit for patients with low or negative PD-L1, with median OS being 35.6 (5.2–66.0) months versus 23.9 (10.1–37.8) months, respectively (*P* = .519) (Fig. 3B).

Cox-regression model analysis showed ICI treatment to be a good prognostic factor for OS in both univariate (HR 0.33; 95% CI 0.16–0.69; *P* = .003) and multivariate analysis (HR 0.35; 95% CI 0.16–0.77; *P* = .009) (Table 4). After adjusted for those not having second line treatment, ICI treatment showed a trend to offer better OS, although the difference was not statically significant in multivariate analysis (HR 0.43; 95% CI 0.18–1.05; *P* = .065) (see Table S4, Supplemental Digital Content 6, which revealed the analysis of overall survival adjusted for patients not receiving second-line therapy).

**Table 4:** Univariate and multivariate analysis of overall survival (OS) (n = 93).

		Univariate		Multivariate	
Variable	n	HR (95% CI)	*P* value^∗^	HR (95% CI)	*P* value^∗^
Age					
Age <65	43	0.86 (0.50–1.50)	.597	1.21 (0.67–2.19)	.523
Age ≥65	50	1		1	
Gender					
Male	71	1.47 (0.74–2.93)	.276	1.80 (0.73–4.45)	.205
Female	22	1		1	
Smoking					
Never smoker	21	1.31 (0.69–2.50)	.417	1.75 (0.75–4.12)	.197
Ever smoker	72	1		1	
ICI treatment					
Yes	23	0.33 (0.16–0.69)	.003	0.35 (0.16–0.77)	.009
No	70	1		1	
*KRAS* subtype					
G12C	34	1.13 (0.65–1.97)	0.666	1.16 (0.66–2.04)	.609
Non-G12C	59	1		1	

CI = confidence interval, HR = hazard ratio, ICI = immune check point inhibitor, *KRAS* = Kirsten rat sarcoma, n = number of patients.

∗ *P* value by Cox regression model.

## 4 Discussion

We have presented the incidence and characteristics of lung cancer patients harboring *KRAS* mutations in Taiwan, where more than half of the lung cancer patients were never-smokers. In our patient group, 21 (22.6%) were never-smokers, with G12C being the most common subtype. Rarely do patients with the *KRAS* mutations also have other co-driver mutations. Patients who never smoked were older in age, more female patients, and with less PD-L1 expression than those who ever or currently smoke. Patients with a TPS ≥50% had higher treatment response rate to ICI than those with a TPS <49%, while smoking status or *KRAS* mutation subtypes did not affect the ICI treatment effect. Most of the *KRAS* mutation patients experienced poor OS (median 13.0 months), regardless of the *KRAS* subtypes. ICI treatment offered survival benefits for these patients, particularly for those with PD-L1 ≥50%.

In western countries, *KRAS* is the most frequent oncogene driver mutation for patients with NSCLC, with an incidence rate of 20% to 25%, and *KRAS* mutation is well known to be associated with smoking behavior.^[2–5]^ In previous Caucasian predominant cohorts, never-smokers represented only 6.4% to 7.1% of all patients with *KRAS*-mutant lung adenocarcinoma, with female patients accounting for >50%.^[2,3]^ However, in East Asian countries, *KRAS* mutations are found in ≤10% of NSCLC patients.^[21]^ In Taiwan, the incidence of such mutations is even lower, dropping down to 5.0% of lung adenocarcinoma patients.^[5]^ Here, high rates of lung cancer in non-smokers may contribute to the lower *KRAS* mutation rate. In the current study, never-smokers accounted for 22.6% of *KRAS* patients. Those who never smoked were older in age and more female cases than those who smoked, which has not been mentioned in previous reports.

*KRAS* mutations are heterogeneous, affecting mainly codons 12, 13, and 61.^[22]^ G12C (39–40%) is the most common substitution in Caucasians, followed by G12V and G12D.^[23]^ Two studies involving Chinese and Korean populations showed similar results, with G12C being the most frequent substitution, followed by G12D and G12V.^[24,25]^ With *KRAS* mutation, never-smokers were more likely than former or current smokers to have a transition mutation (G→A), compared with transversion mutations which are known to be smoking related (G→T or G→C).^[26]^ In Caucasian smokers, the most frequent oncogenic substitution is G12C, which is found in 41% to 43% of *KRAS*-mutant NSCLC; whereas in non-smokers or light-smokers, both G12C and G12D have been reported more common.^[2,23]^ In our study, G12C was the most common *KRAS* mutation in all of our patient (36.6%) and in ever-smokers (38.9%). In never-smokers, we found G12C (28.6%), G12D (23.8%), and G12V (28.6%) as being most prevalent. As we found similar G12C mutation rates regardless of smoking habits, never-smokers should not be excluded in any future studies on G12C inhibitors.

In NSCLC*, KRAS* mutations usually indicate a poor prognosis. Gow et al^[27]^ analyzed driver mutations in 888 Asian lung cancer patients. Compared with stage-IIIB/IV lung cancer patients with pan-negative driver mutations (OS 12.3 months), those with mutations of *EGFR* (OS 22.5 months) or *ALK* (OS 21.9 months) experienced better OS; while *KRAS* mutations (OS 6.4 months) were associated with poor OS. *KRAS* mutation subtypes may contribute to different outcomes, but these results are controversial.^[12]^ In a post hoc analysis including 300 patients with *KRAS* mutations in 4 clinical trials regarding adjuvant chemotherapy, the presence of codon 13 mutations was associated with worse OS. There were no differences in OS and disease-free survival across the different codon 12 mutations.^[4]^ Aredo et al^[28]^ included 186 NSCLC patients with *KRAS* mutations in stages I to IV and found that *KRAS* G12D mutations were associated with poor OS, as were STK11 co-mutations. Another study, which included patients with lung cancer harboring *KRAS* mutations in advanced stages, reported that those with *KRAS* G12C mutation appeared to have longer progression-free survival after undergoing first-line chemotherapy.^[13]^ A cohort in the United States suggested that none of the *KRAS* subtypes impact survival, though a positive PD-L1 status revealed a worse outcome in patients with *KRAS* G12C mutation.^[12]^ In our study, there were no survival differences across the *KRAS* mutation subtypes (Fig. 2 and Table 4).

In addition to chemotherapy, NSCLC patients with *KRAS* mutations may benefit from ICI. In a meta-analysis, when compared with docetaxel, ICI improved OS in patients with *KRAS* mutant NSCLC.^[29]^ Other studies revealed a similar or better ICI treatment efficacy for NSCLC patients harboring *KRAS* mutations than those with *KRAS* wild type.^[11,30–32]^ Different *KRAS* subtypes have been shown correlated to PD-L1 expression levels. Judd et al^[23]^ found higher TPS in patients with *KRAS* G12C than other subtypes, and a higher tumor mutation burden in patients with G13 subtypes. The results of IMMUNOTARGET registry suggested that the PD-L1 expression levels impact the effects of ICI on patients with *KRAS* mutations.^[10]^ In their study, 271 patients harboring *KRAS* mutations were included, and showed a response rate 26% to ICI. When the PD-L1 expression was positive, patients experienced longer PFS after ICI treatments. Another study, enrolling 162 NSCLC patients with *KRAS* mutations, showed a trend which ICI offered a better response rate and PFS when the PD-L1 was higher, though the results were not statistically significant.^[11]^ In addition, co-mutations may predict the different clinical benefits of ICI. Concomitant pathogenic mutations have been identified in *KRAS* mutant NSCLC, with *TP53* (39–53%) and *STK11* (14–37%) the most common, while *EGFR* mutations (0–1%), *BRAF* mutations (1–5%), and *ALK* fusions (0.5%) were rarely found.^[23,33]^ Co-mutation of *KRAS* and *TP53* shows an increased PD-L1 expression.^[28]^ In the SU2C cohort, objective response rates to PD-1 blockade differ amongst the co-mutations with *KRAS* and *STK11* (7.4%), co-mutations with *KRAS* and *TP53* (35.7%), and *KRAS* only (28.6%) subgroups.^[34]^ In other words, *KRAS*/*STK11* mutant tumors exhibit a weak immune-tumor micro-environment, while those with *KRAS*/*TP53* exhibit an immunogenic micro-environment.^[22]^ In our study, concomitant mutations with *KRAS* mutations were identified in *EGFR* mutations (1.1%), *ALK* fusions (1.1%), and *BRAF* V600E mutations (1.1%). In addition, when PD-L1 data were available, 66.0% showed positive PD-L1, while 38.3% had high PD-L1 expressions (TPS ≥50%). The *KRAS* mutation subtypes showed no correlation with the PD-L1 results, while ever-smokers were more likely to show high PD-L1 expressions. The response rate to ICI was 21.7% in the current study. Patients with TPS ≥50% experienced higher response rates (45.5%) to ICI. Moreover, patients receiving ICI had longer OS (median 35.6 vs 9.8 months), particularly for whom with TPS ≥50% (median OS not-reached vs 8.4 months).

There were several limitations in our present study. First, the study was conducted in Taiwan, a region with high rates of both *EGFR* mutants and non-smoking lung cancer patients.^[5]^ Our data may not be generalized for other countries where more lung cancer patients are smokers. Second, our study was retrospective with inevitable biases. Third, the sample size was limited, especially for patients with available PD-L1 expression results. Forth, we did not include NSCLC patients with *KRAS* wild type. As a result, it was not capable to compare between patients with and without *KRAS* mutations, for the response rate and survival benefit of ICI treatment. Though notorious for being an “undruggable” mutation, *KRAS* mutation became a therapeutic target. Sotorasib (AMG 510), an experimental small molecule which irreversibly binds G12C, has been granted breakthrough therapy designation by US FDA for the treatment of patients with locally advanced or metastatic NSCLC with *KRAS G12C* mutation. After receiving the target dose of 960 mg once daily, 32.2% patients achieved the objective response, while 88.1% achieved disease control, with a median PFS of 6.3 months.^[9,35]^ For advanced NSCLC patients with *EGFR* mutations, after *EGFR*-TKI treatment, smokers had shorter progression free survival when compared with non-smokers.^[36]^ This negative effect on the survival of smokers may be explained by smoking-induced cytochromes CYP1A1/1A2, which presumably alter anti-*EGFR* erlotinib pharmacokinetics.^[37]^ Additionally, not only *EGFR*-TKI, but the efficacy of ALK inhibitors also appeared to be reduced due to smoking behavior.^[38]^ Unlike *EGFR* and *ALK* mutations, which are detected in higher proportions in non-smokers and former smokers, *KRAS* mutations are more prevalent amongst smokers. However, our study found that never-smokers accounted for 17.6% of all G12C patients. Whether smoking behavior affects the efficacy of AMG510 warrants further investigation. Additional research is needed to elucidate whether smoking is associated with clinical outcomes in *KRAS*-mutant NSCLC patients.

## 5 Conclusion

To conclude, in Taiwan, more than one-fifth of *KRAS* mutations in NSCLC were never-smokers, who were older in age, more female patients, and with lower PD-L1 expression levels. The G12C mutation rate being the most common in both never- and ever-smokers. For NSCLC patients with *KRAS* mutations, higher PD-L1 expression predicted a better ICI treatment response. Patients receiving ICI treatment had longer OS, particularly for those with a TPS ≥50%.

## Author contributions

**Conceptualization:** Gee-Chen Chang, Jia-Jun Wu, Kun-Chieh Chen, Po-Hsin Lee.

**Data curation:** Jia-Jun Wu, Kun-Chieh Chen, Kuo-Hsuan Hsu, Po-Hsin Lee, Zhe-Rong Zheng.

**Formal analysis:** Gee-Chen Chang, Jeng-Sen Tseng, Yen-Hsiang Huang.

**Investigation:** Jia-Jun Wu, Zhe-Rong Zheng.

**Methodology:** Gee-Chen Chang, Jeng-Sen Tseng, Jia-Jun Wu, Kun-Chieh Chen, Po-Hsin Lee, Sung-Liang Yu, Tsung-Ying Yang, Yen-Hsiang Huang.

**Project administration:** Gee-Chen Chang, Kun-Chieh Chen, Kuo-Hsuan Hsu.

**Resources:** Gee-Chen Chang, Sung-Liang Yu, Tsung-Ying Yang.

**Software:** Sung-Liang Yu, Yen-Hsiang Huang.

**Supervision:** Gee-Chen Chang, Jeng-Sen Tseng, Kun-Chieh Chen, Tsung-Ying Yang.

**Validation:** Jeng-Sen Tseng, Kun-Chieh Chen, Tsung-Ying Yang.

**Visualization:** Kuo-Hsuan Hsu, Sung-Liang Yu, Yen-Hsiang Huang.

**Writing – original draft:** Jia-Jun Wu, Po-Hsin Lee, Zhe-Rong Zheng.

**Writing – review & editing:** Gee-Chen Chang, Kun-Chieh Chen.

## Supplementary Material

Supplemental Digital Content

## Supplementary Material

Supplemental Digital Content

## Supplementary Material

Supplemental Digital Content

## Supplementary Material

Supplemental Digital Content

## Supplementary Material

Supplemental Digital Content

## Supplementary Material

Supplemental Digital Content
