# Supplementary material for: Characteristics and immune checkpoint inhibitor effects on non-smoking non-small cell lung cancer with KRAS mutation: A single center cohort (STROBE-compliant)
Source: Medicine (Baltimore). 2022 Jun 17;101(24):e29381. doi: 10.1097/MD.0000000000029381 (PMC9276274; doi:10.1097/MD.0000000000029381)

**Supplemental Digital Content Legends**

Figure S1., Supplemental digital content 2, Flow chart of patient enrollment. A total of 2,932 patients were tested for five driver genes (*EGFR*, *KRAS*, *HER2*, *BRAF*, and *ALK*) from a single center, with 151 (5.2%) patients having *KRAS* mutation. After excluding 58 patients, 93 patients were analyzed for both clinicopathological characters and treatment outcomes.


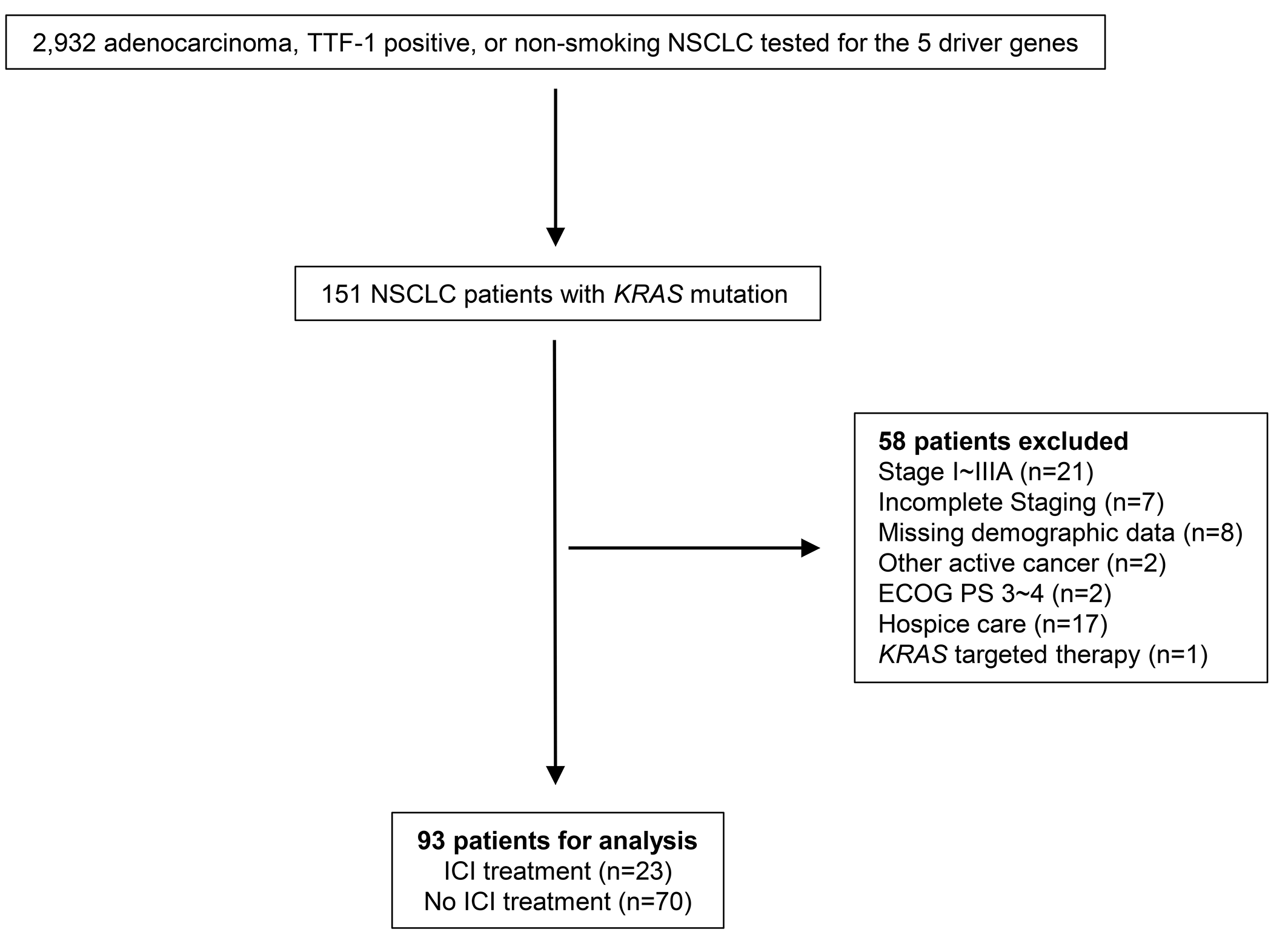

Supplement: Supplemental Digital Content [file medi-101-e29381-s001.doc]
