# Supplementary material for: Characteristics and immune checkpoint inhibitor effects on non-smoking non-small cell lung cancer with KRAS mutation: A single center cohort (STROBE-compliant)
Source: Medicine (Baltimore). 2022 Jun 17;101(24):e29381. doi: 10.1097/MD.0000000000029381 (PMC9276274; doi:10.1097/MD.0000000000029381)
Supplement: Supplemental Digital Content [file medi-101-e29381-s003.docx]

**Supplemental Digital Content 3**

| Table S2. Smoking behavior across different genders and *KRAS* subtypes | | | |
| --- | --- | --- | --- |
|  | Never smoker | Ever smoker | *p* value* |
| Gender, number (%) |  |  |  |
| Male | 7 (9.9) | 64 (90.1) | <0.001 |
| Female | 14 (63.6) | 8 (36.4) |  |
| *KRAS* subtype, number (%) |  |  |  |
| G12C | 6 (28.6) | 28 (38.9) | 0.803 |
| G12D | 5 (23.8) | 15 (20.8) |  |
| G12V | 6 (28.6) | 11 (15.3) |  |
| G12A | 3 (14.3) | 9 (9.7) |  |
| G12S | 0 (0.0) | 3 (4.2) |  |
| G13C | 0 (0.0) | 2 (2.8) |  |
| G13D | 1 (4.8) | 1 (1.4) |  |
| G13A | 0 (0.0) | 1 (1.4) |  |
| G13V | 0 (0.0) | 1 (1.4) |  |
| G12C + G12V | 0 (0.0) | 1 (1.4) |  |
| ^*^Probability value by Chi-square test. | | | |
