# Supplementary material for: Characteristics and immune checkpoint inhibitor effects on non-smoking non-small cell lung cancer with KRAS mutation: A single center cohort (STROBE-compliant)
Source: Medicine (Baltimore). 2022 Jun 17;101(24):e29381. doi: 10.1097/MD.0000000000029381 (PMC9276274; doi:10.1097/MD.0000000000029381)

**Supplemental Digital Content Legends**

Figure S2., Supplemental digital content 4, Distribution of PD-L1 expression (A) and response to ICI treatment (B). Ever smokers were related to a higher PD-L1 expression (*p░=░0.038 in Chi-square test, p░=░0.039 in univariate and p░=░0.031 in multivariate analysis through the logistic regression model to predict high PD-L1 expression). Patients with high PD-L1 expression had a better response rate to ICI treatment (#p░=░0.035 by Chi-square test).

*KRAS*, Kirsten rat sarcoma; PD-L1, programmed death-ligand 1.


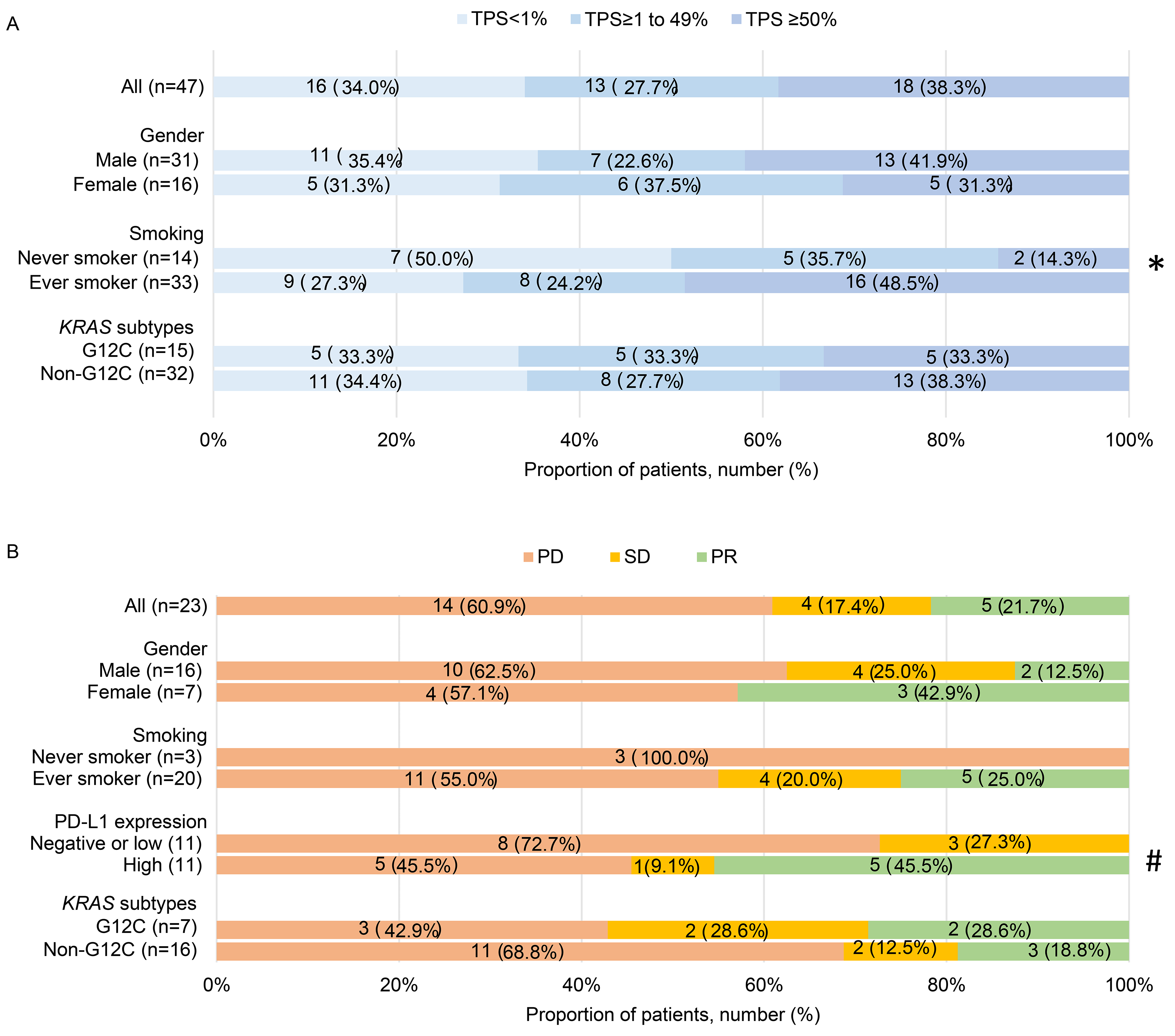

Supplement: Supplemental Digital Content [file medi-101-e29381-s004.doc]
