# Supplementary material for: Characteristics and immune checkpoint inhibitor effects on non-smoking non-small cell lung cancer with KRAS mutation: A single center cohort (STROBE-compliant)
Source: Medicine (Baltimore). 2022 Jun 17;101(24):e29381. doi: 10.1097/MD.0000000000029381 (PMC9276274; doi:10.1097/MD.0000000000029381)
Supplement: Supplemental Digital Content [file medi-101-e29381-s005.docx]

**Supplemental Digital Content 5**

| Table S3. Patients who received immunotherapy (n=23) | | | | | | | | | | | |
| --- | --- | --- | --- | --- | --- | --- | --- | --- | --- | --- | --- |
| Case | Gender | Age | Smoking status | Stage | Histology | Regimen | Line of treatment of ICI | Response | ECOG PS | PD-L1, (%) | KRAS subtypes |
| **1** | F | 74 | E | IVB | Ad | P | 1 | PR | 1 | $\geq$50 | G12V |
| **2** | M | 71 | E | IVA | Ad | C | 2 | PR | 1 | $\geq$50 | G12C |
| **3** | F | 73 | N | IVA | Ad | P | 1 | PD | 2 | 1~50 | G12D |
| **4** | M | 51 | E | IVA | Ad | P | 4 | PD | 1 | $\geq$50 | G12C + G12V |
| **5** | M | 58 | E | IVA | Ad | Ni | 6 | PD | 1 | <1 | G12D |
| **6** | M | 56 | E | IIIB | Ad | Ni | 4 | PD | 1 | <1 | G12V |
| **7** | M | 53 | E | IIIB | Ad | P | 2 | SD | 1 | <1 | G12C |
| **8** | M | 56 | E | IVB | Ad | P | 4 | PD | 1 | $\geq$50 | G13D |
| **9** | M | 56 | E | IVB | Ad | P | 3 | PR | 1 | $\geq$50 | G12D |
| **10** | F | 50 | E | IV | Ad | Ni | 3 | PD | 1 | <1 | G12C |
| **11** | F | 72 | N | IV | Ad | A | 3 | PD | 1 | 1~50 | G12V |
| **12** | F | 60 | N | IV | Ad | P | 3 | PD | 1 | 1~50 | G12C |
| **13** | M | 57 | E | IVB | Ad | P | 1 | PD | 1 | 1~50 | G12C |
| **14** | F | 50 | E | IVB | Ad | P | 2 | PR | 1 | $\geq$50 | G12C |
| **15** | M | 57 | E | IVB | Ad | Ni | 3 | PD | 1 | $\geq$50 | G12D |
| **16** | M | 71 | E | IVA | Ad | P | 1 | SD | 1 | $\geq$50 | G12C |
| **17** | M | 58 | E | IIIC | Ad | Ni | 6 | SD | 1 | <1 | G12V |
| **18** | F | 68 | E | IVB | Ad | P | 2 | PR | 1 | 100% | G12A |
| **19** | M | 67 | E | IVB | Ad | P | 2 | PD | 1 | 50% | G12D |
| **20** | M | 49 | E | IVB | Ad | At | 5 | PD | 1 | 10% | G12S |
| **21** | M | 49 | E | IVB | Ad | P | 1 | SD | 1 | 40% | G12D |
| **22** | M | 51 | E | IVB | Ad | P | 2 | PD | 1 | 70% | G12A |
| **23** | M | 47 | E | IIIC | Ad | At | 2 | PD | 1 | Not test | G12D |
| M, male; F, female; N, never smoker; E, ever smoker; ECOG PS, Eastern Cooperative Oncology Group performance status; Ad, adenocarcinoma; SQUA, squamous cell carcinoma; At, atezolumumab; C, cemiplimab; Ni, nivolumab; P, pembrolizumab; *KRAS*, Kirsten rat sarcoma; PD-L1, programmed death-ligand 1. | | | | | | | | | | | |
