# Supplementary material for: Characteristics and immune checkpoint inhibitor effects on non-smoking non-small cell lung cancer with KRAS mutation: A single center cohort (STROBE-compliant)
Source: Medicine (Baltimore). 2022 Jun 17;101(24):e29381. doi: 10.1097/MD.0000000000029381 (PMC9276274; doi:10.1097/MD.0000000000029381)
Supplement: Supplemental Digital Content [file medi-101-e29381-s006.docx]

**Supplemental Digital Content 6**

| Table S4. Univariate and multivariate analysis of overall survival (OS) (adjusted for patients not receiving second-line therapy) (n=60) | | | | | |
| --- | --- | --- | --- | --- | --- |
|  |  | Univariate | | Multivariate | |
| Variable | n | HR (95% CI) | *p* Value^a^ | HR (95% CI) | *p* Value^a^ |
| Age |  |  |  |  |  |
| Age $<$ 65 | 29 | 0.75 (0.38 – 1.49) | 0.418 | 1.06 (0.50 – 2.26) | 0.872 |
| Age $\geq$ 65 | 31 | 1 |  | 1 |  |
| Gender |  |  |  |  |  |
| Male | 46 | 1.61 (0.67 – 3.90) | 0.289 | 1.79 (0.55 – 5.82) | 0.331 |
| Female | 14 | 1 |  | 1 |  |
| Smoking |  |  |  |  |  |
| Never smoker | 12 | 1.21 (0.53 – 2.79) | 0.651 | 1.64 (0.54 – 4.95) | 0.384 |
| Ever smoker | 48 | 1 |  | 1 |  |
| ICI treatment |  |  |  |  |  |
| Yes | 23 | 0.38 (0.17 – 0.82) | 0.014 | 0.43 (0.18 – 1.05) | 0.065 |
| No | 37 | 1 |  | 1 |  |
| *KRAS* subtype |  |  |  |  |  |
| G12C | 21 | 1.38 (0.70 – 2.70) | 0.352 | 1.34 (0.67 – 2.69) | 0.409 |
| Non-G12C | 39 | 1 |  | 1 |  |
| ^a^*p* value by Cox regression model.  CI, confidence interval; HR, hazard ratio; ICI, immune check point inhibitor; *KRAS*, Kirsten rat sarcoma; n, number of patients. | | | | | |
